# Icotinib combined with rapamycin in a renal transplant recipient with epidermal growth factor receptor-mutated non-small cell lung cancer: A case report

**DOI:** 10.3892/ol.2013.1657

**Published:** 2013-11-05

**Authors:** QIONG ZHAO, YINA WANG, YEMIN TANG, LING PENG

**Affiliations:** Department of Thoracic Oncology, The First Affiliated Hospital, School of Medicine, Zhejiang University, Hangzhou, Zhejiang 310003, P.R. China

**Keywords:** lung cancer, kidney transplantation, icotinib, rapamycin, interstitial pneumonitis

## Abstract

As kidney transplant recipients are at increased risk of developing cancer, regular monitoring should be undertaken to monitor the balance between immunosuppression and graft function and to identify malignancy. The present study reports the outcome of the treatment of adenocarcinoma of the lung (T1aN0M1a, stage IV) using the molecular-targeted therapy, icotinib, in a 66-year-old male renal transplant patient receiving rapamycin and prednisolone as ongoing renal immunosuppressive therapy. An initial partial response to icotinib was achieved, and graft function remained good. However, the patient subsequently developed interstitial pneumonitis. The plasma concentrations of rapamycin and icotinib were within the normal ranges, which excluded the possibility of a pharmacokinetic drug interaction and indicated that the interstitial pneumonitis was likely to be associated with the side-effects of icotinib. Drug therapy was discontinued and the patient underwent a segmentectomy. Tacrolimus was administered for ongoing renal graft immunosuppression. To the best of our knowledge, this is the first report of the concomitant administration of icotinib and rapamycin in post-transplant *de novo* lung cancer. It is also the first report of interstitial pneumonitis associated with icotinib in a post-transplant patient.

## Introduction

Organ transplant recipients are at increased risk of developing malignancies, with an overall cancer incidence estimated at 1,375 cases/100,000 person-years (1). The incidence of lung cancer has been shown to be approximately two-fold higher during the first three years after kidney transplantation than in the general population, with the majority of patients being diagnosed at an advanced stage (2). The treatment of lung cancer in transplant recipients is complicated, as the complex clinical situation and immunosuppressive drug administration often require conflicting therapeutic approaches. The present study reports the first case of stage IV *de novo* lung cancer developing four months after renal transplantation. The patient developed drug-induced interstitial pneumonitis while receiving immunosuppressive drugs and oral-targeted therapy concomitantly. Written informed consent was obtained from the patient.

## Case report

The patient was a 66-year-old male who presented with a history of chronic glomerulonephritis of >30 years and had been receiving dialysis for five years. In April 2010, the patient underwent allogenic renal transplantation followed by immunosuppressive therapy with cyclosporin A (CsA; 50 mg twice daily), mycophenolate mofetil (MMF; 500 mg twice daily) and prednisolone (5 mg twice daily). The serum creatinine level remained between 90 and 110 μmol/l and there were no episodes of acute rejection. The patient had stopped smoking two years prior to transplantation, but had a 40-year history of smoking 20 cigarettes/day. There was no family history of lung cancer. The patient did not complain of coughing, expectoration, hemoptysis or chest pain prior to kidney transplantation, and a chest X-ray revealed no signs of abnormality.

**Figure 1:**
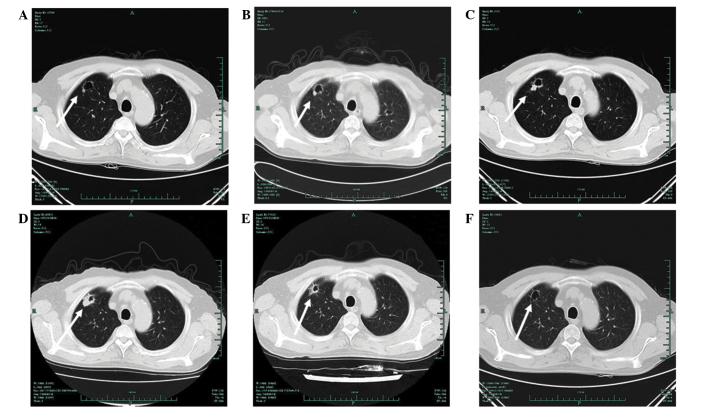
Chest computed tomography (CT) findings showing evidence of a tumor response. Chest CT images showed a tumor response prior to and after the patient received icotinib. (A) The first appearance of a nodule on August 12, 2010, at four months post-kidney transplant. (B) On February 9, 2011, the nodule remained stable as previously. (C) On October 13, 2011, CT imaging showed a marginal increase in the size of the nodule. (D) On February 15, 2012 (prior to treatment with icotinib), the nodule was pathologically diagnosed as adenocarcinoma. (E) Imaging results on March 12, 2012, following one week of treatment with icotinib. (F) Imaging results on August 7, 2012, following five months of treatment with icotinib. The patient met the Response Evaluation Criteria in Solid Tumors (RECIST) for a partial response. The arrows indicate the tumor site.

**Figure 2:**
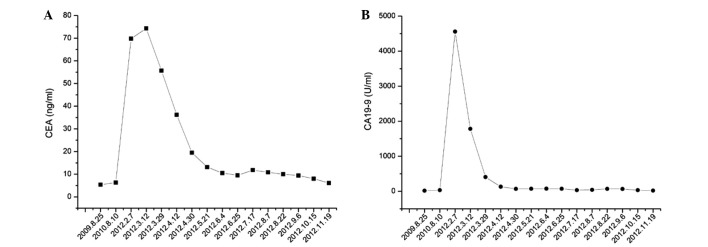
Serum tumor markers prior to and following icotinib treatment. Serum (A) carcinoembryonic antigen (CEA) and (B) CA19-9 levels decreased following treatment with icotinib.

A follow-up chest X-ray in August 2010 showed evidence of a suspicious nodule in the right upper lobe of the lung. A chest computed tomography (CT) scan revealed a 6-mm diameter nodule, with a surrounding cavity and fibrous lesions (Fig. 1). Bronchoscopic biopsy, abdominal B ultrasound, cranial MRI and bone scans excluded distant metastasis. The carcinoembryonic antigen (CEA) levels were 5.7 ng/ml (normal range, 0–5 ng/ml) and the carbohydrate/cancer antigen 19–9 (CA19–9) level was 15.7 U/ml (normal range, 0–37 U/ml) (Fig. 2). All other serum tumor markers were within the normal range. A follow-up chest CT in December 2010 showed that the nodule had become enlarged, and identified multiple ipsilateral subpleural nodules, all of which were <5 mm in diameter. Based on these findings, the immunosuppression protocol was switched to rapamycin (0.5 mg once daily), MMF (500 mg twice daily) and prednisolone (5 mg once daily).

Regular CT follow-up and serum tumor marker tests performed every three months indicated that the nodule and serum tumor markers remained stable until the end of 2011. In February 2012, the CEA level had increased to 69.8 ng/ml and the CA19–9 level had increased to 4,559 U/ml. Chest CT imaging on February 15, 2012, revealed further significant enlargement of the nodule, with ipsilateral multiple subpleural nodules. An abdominal contrast CT was performed to exclude primary tumors of the digestive tract.

A CT-guided tumor biopsy enabled the nodule to be pathologically diagnosed as adenocarcinoma, stage IV, T1aN0M1a (3). Molecular testing undertaken using the polymerase chain reaction-amplification refractory mutation system (PCR-ARMS) indicated that the patient harbored a deletion in exon 19 and an L858R point mutation in exon 21, but there was no evidence of a T790M mutation in exon 20.

On March 1, 2012, the patient was scheduled to receive molecular-targeted therapy with oral icotinib (125 mg three times a day) for six days. However, the drug was discontinued after five days due to personal reasons. A chest CT on March 12, 2012, indicated a substantial remission of the lung nodule, with no change in the subpleural nodules. The patient restarted treatment with icotinib (125 mg three times a day) on March 12, 2012. On March 29, 2012, the CEA level had dropped to 55.7 ng/ml and the CA19-9 level had dropped to 406.6 U/ml. A follow-up chest CT scan subsequent to more than one month of icotinib treatment showed evidence of a further decrease in the size of the pulmonary nodule (Fig. 1), and a partial response (PR) was evaluated according to the Response Evaluation Criteria in Solid Tumors (3). MMF was discontinued, and rapamycin (0.75 mg once daily) and prednisolone (5 mg once daily) were used as the ongoing immunosuppressive protocol. By April 30, 2012, the CEA level had decreased to 19.5 ng/ml and the CA19-9 level to 69.4 U/ml (Fig. 2).

The patient received icotinib for a further five months with a maintained RECIST PR for all disease parameters. Icotinib was extremely well tolerated, with only grade 2 skin toxicity appearing one week after the onset of treatment.

**Figure 3:**
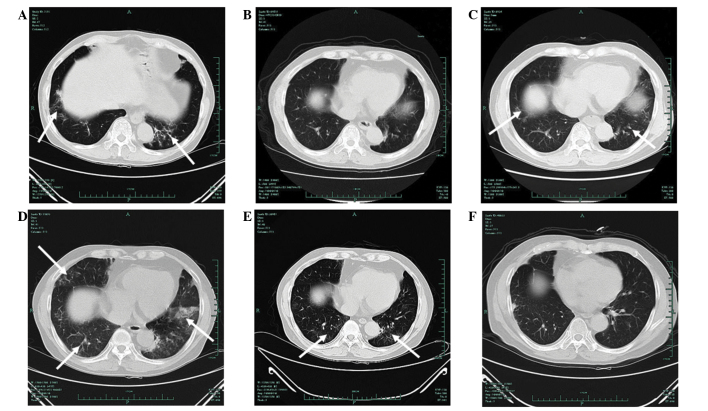
Computed tomography (CT) evidence of interstitial pneumonitis. (A) On October 13, 2011, the CT image showed for the first time scattered, patchy shadows in the two lower lobes of the lung. (B) On February 15, 2012, no shadows were detected. (C) CT findings on May 12, 2012, three months after the start of icotinib. (D) CT findings on August 7, 2012, five months after the start of icotinib. (E) CT findings on October 15, 2012, two months after the patient discontinued rapamycin and icotinib. (F) CT findings on November 10, 2012, one month after the patient underwent a segmentectomy. The arrows indicate the site of the interstitial lung disease.

A chest CT on August 7, 2012, showed bilateral patchy and diffuse interstitial infiltrates, which were signs of interstitial pneumonitis (Fig. 3). The patient did not complain of any discomfort, and infectious causes and other pulmonary diseases were excluded. A review of the previous chest CT images identified an unnoticed, transient interstitial pneumonitis on October 13, 2011 (Fig. 3). Lung auscultation identified wheezing, and pulmonary function tests indicated a modest decrease in diffusing capacity.

**Figure 4:**
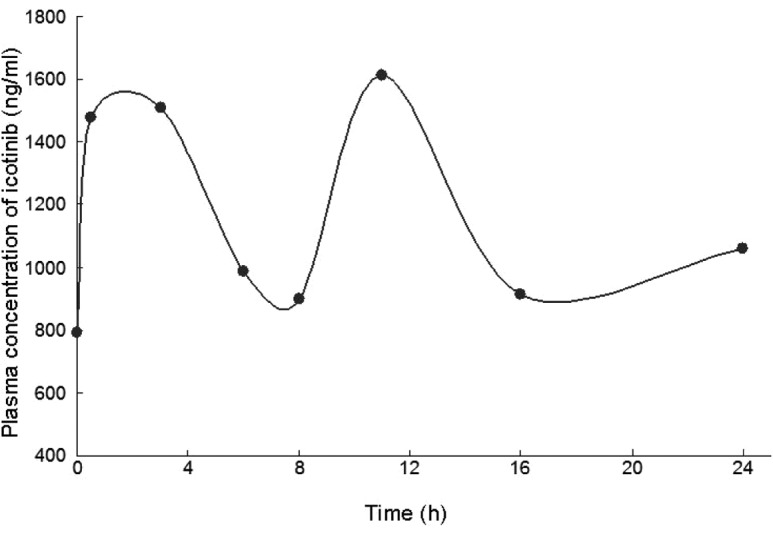
Plasma concentration of icotinib. Eight time points (0, 0.5, 3, 6, 8, 11, 16 and 24 h post-dose) were selected to determine the plasma concentration of icotinib (125 mg TID) by high-performance liquid chromatography tandem mass spectrometry. The resulting plasma concentration time profile was similar to that in the published phase I trial of icotinib (3).

The plasma concentrations of icotinib were determined by Beta Pharma Co., Ltd. (Hangzhou, China) using a high-performance liquid chromatography method. The results (Fig. 4) showed concentrations similar to those reported in the published phase I trial of icotinib (4), indicating that the interstitial pneumonitis was not due to the increase in icotinib plasma concentration. The pharmacokinetic data from the patient was also similar to that estimated in the phase I population (Table I).

Based on these findings, the patient was diagnosed with icotinib-associated interstitial pneumonitis. Icotinib and rapamycin were discontinued, and methylprednisolone tablets (8 mg twice daily) were administered to treat interstitial pneumonitis. A CT scan on August 22, 2012, showed a radiological improvement in the interstitial pneumonitis. The immunosuppressive drug treatment was changed to tacrolimus (FK506; 1 mg twice daily).

On October 17, 2012, the patient underwent a video-assisted segmentectomy to remove the apicoposterior segment of the right upper lobe. Surgical biopsy confirmed the pathological diagnosis of adenocarcinoma; the immunohistochemistry results showed that the specimen was CK7^+^, P63^−^ and thyroid transcription factor-1^+^. Molecular testing by PCR-ARMS confirmed that the patient harbored a deletion in exon 19, but not in exon 21 and 20.

At the time that this report was written, the patient was being followed up by quarterly chest CT scans and serum tumor marker testing. The most recent concentration of CEA on November 19, 2012, was 6.1 ng/ml, and the CA19-9 levels were 19.1 U/ml. The concentration of tacrolimus was maintained within the optimal therapeutic range of 3.1–4.4 ng/ml. The patient’s renal function also remained good up to this time.

## Discussion

Immunosuppressive agents inhibit immune responsiveness and in the long-term have the potential to accelerate tumor growth and metastasis. Immunosuppressive agents, such as azathioprine, have been shown to exert a direct oncogenic effect by causing chromosomal breakdown (5). CsA and MMF have been associated with post-transplant malignancy, but the cancer incidence with MMF is lower than that with CsA (6).

Rapamycin is an inhibitor of the mammalian target of rapamycin (mTOR). Rapamycin acts as an immunosuppressant, but also possesses antiproliferative activity, which may be useful in post-transplant patients at increased risk of malignancy (7). Early withdrawal of CsA and a switch to mTOR inhibitors, such as rapamycin and everolimus, have been shown to reduce the risk of cancer in renal transplant patients (8,9). Data from clinical trials and large registries indicate that the incidence of *de novo* malignancies is less frequent among patients receiving mTOR inhibitors than among those receiving other forms of immunosuppressive therapy (10).

The modulation, switch or discontinuation of immunosuppressive drugs in post-transplant patients has to be made on a case-by-case basis. Upon the identification of a *de novo* lung nodule in the present study patient, CsA was discontinued and the dose of MMF was reduced and then discontinued. Subsequent to being diagnosed with lung cancer, the patient was switched to rapamycin and prednisolone. The immunosuppressive effect of rapamycin, in combination with its antitumor effects, make this drug an attractive treatment for post-transplant malignancies.

On October 13, 2011, the patient developed transient, asymptomatic and unnoticed interstitial pneumonitis, which was considered to be the consequence of the rapamycin treatment. It has previously been reported that as many as one in six patients taking mTOR inhibitors develop reversible interstitial pneumonitis (11). Sirolimus pulmonary toxicity has also been reported in renal transplant patients (12–14). In approximately half of the cases this develops within six months of starting treatment. The exact pathogenic mechanism of sirolimus-induced pulmonary toxicity is not known, but it has been reported to be dose-dependent and male-dominant (13).

The ongoing interstitial pneumonitis detected in the present patient after five months of icotinib treatment was mainly due to the administration of this drug. Radiological improvement subsequent to the cessation of icotinib treatment indicated a causal correlation.

The incidence of drug-induced lung disease by molecular-targeted therapy varies among different drugs. The incidence of interstitial lung disease (ILD) in patients receiving erlotinib is reported to be between 1 and 3.8% (14–17), while the incidence with gefitinib is between 1 and 8.3% (18–22). A study in a population with a high co-incidence of pulmonary disease proposed that the mechanism for developing epidermal growth factor receptor (EGFR) tyrosine kinase inhibitor (TKI)-induced ILD was most likely related to a decrease in alveolar regeneration (23). This process was shown to be normally regulated by EGFR. The treatment of drug-induced interstitial pneumonitis includes discontinuation of the suspect drug, administration of high-dose corticosteroids and mechanical ventilation. Resuming administration of the previous drug following the resolution of symptoms may lead to recurrence of ILD (24).

The potential synergy between the mTOR inhibitor, rapamycin, and icotinib may have contributed to the rapid remission of the tumor in the present case. Studies in animal models have demonstrated *in vitro* synergistic effects between rapamycin and erlotinib in non-small cell lung cancer (NSCLC) and pancreatic, colon and breast tumors (25,26). Rapamycin has also been shown to be effective in clinical trials with EGFR TKIs in the treatment of glioblastoma and renal cell carcinoma (27,28). Other mTOR inhibitors, such as everolimus, have been administered in combination with EGFR TKIs in NSCLC (29), providing new insights for the treatment of post-transplant lung malignancies.

**Figure 5:**
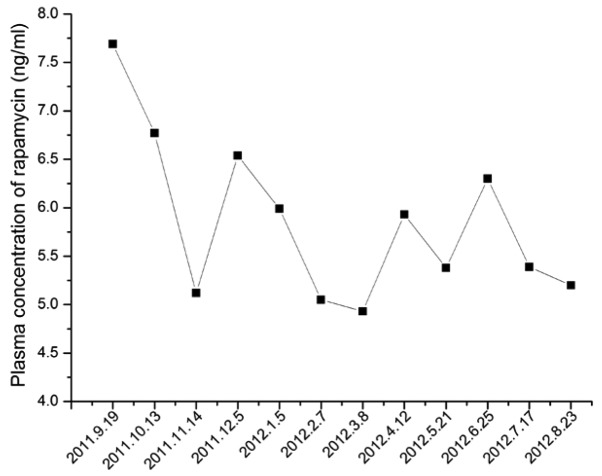
Plasma concentration of rapamycin. The patient received rapamycin (50 mg QD) for seven months, followed by rapamycin (0.5 mg QD) for one month in combination with icotinib (125 mg TID). This was followed by rapamycin (0.75 mg QD) for five months in combination with icotinib (125 mg TID). Plasma levels of rapamycin remained within the predefined optimal range.

The metabolism of icotinib is undertaken mainly by CYP3A4 and CYP2C19 (unpublished data). Thus, the combination of icotinib with rapamycin [a known CYP3A4 substrate (30)] may have increased the potential for unexpected side-effects. However, there are no published data on the pharmacodynamics and interactions between the mTOR inhibitor and icotinib. Similarly, there are no clinical trials on the safety and efficacy of this combination in NSCLC. In the present case, we speculate that the drug-induced interstitial pneumonitis was not due to the interaction of the two drugs, but that it resulted from the individual pulmonary toxicity of icotinib, since the plasma concentrations of icotinib and rapamycin each remained within their optimal ranges (Fig. 5).

Post-transplant lung cancer is often associated with non-smokers and an adenocarcinoma histology (31). The present patient was a former smoker and the histology of the tumor was of an adenocarcinoma. Prior to the pathological diagnosis, the tumor had gradually developed from stage I to stage IV disease. Molecular testing confirmed that the patient harbored a deletion in exon 19 and an L858R point mutation in exon 21, which indicated that EGFR TKIs may provide some benefit. Icotinib (4,32) is an oral EGFR TKI that has been approved by the Chinese State Food and Drug Administration (FDA) for the treatment of advanced NSCLC. In total, >5,000 Chinese patients have received this drug in the last year. However, the present study is the first report of the administration of icotinib in a post-transplant patient.

The patient achieved a PR subsequent to receiving icotinib for six days. Research has shown that patients with double-activating mutations in exon 19 and 21 account for ~3.4% of unselected NSCLC patients of Chinese origin. These patients tend to respond well to TKIs, as the sensitivity of double-mutated EGFR TKIs is higher than that observed among patients with single mutations (33). However, the presence of double mutations is not only associated with higher clinical response rates, but may also contribute to the high incidence of pulmonary toxicity (34).

Tumors that develop following kidney transplantation are generally more malignant, more poorly differentiated and carry a worse prognosis compared with corresponding tumors in other populations. Tumor screening and early diagnosis are therefore essential prior to and following transplantation. Measures to reduce the risk of post-transplant malignancies, including CT screening for lung cancer and smoking cessation, should be recommended for transplant recipients.

Rapamycin and other mTOR inhibitors are associated with unique side-effects (7), the majority of which are dose-related. This means that the monitoring of drug levels should be routinely undertaken in patients receiving icotinib as a targeted therapy.

To the best of our knowledge, this is the first report of a concomitant administration of icotinib and rapamycin. This combination of EGFR-TKIs and mTOR inhibitors may provide an attractive regimen for the subset of patients that develops advanced NSCLC following kidney transplantation. However, consideration of the unique side-effects associated with this combined regimen strategy requires further evaluation in randomized double-blind trials. An improved understanding of drug-induced ILD is also required, including more reliable data on the incidence of events associated with different treatments and identification of the risk factors for this type of ILD. Clinicians should remain aware of the possibility of drug-induced pulmonary toxicity when using mTOR inhibitors in combination with icotinib.

## Figures and Tables

**Table I:** Comparison of the pharmacokinetics data from the present patient and data obtained from a phase I trial of icotinib.

	Phase I trial (TID)			Patient (TID)
		
Parameter, h	125 mg	250 mg	375 mg	125 mg
T_max_	2.250	2.500	2.890	3.000
C_max_	2.100	3.910	4.400	1.613
C_min_	1.020	2.090	2.310	0.957
AUC_last_	34.600	40.600	42.600	27.897

TID, three times a day; T_max_, time to maximum plasma concentration; C_max_, maximum concentration; C_min_, minimum concentration; AUC_last_, area under the plasma concentration-time profile from time zero to the time of the last quantifiable concentration.
